# Understanding of Adsorption and Desorption Mechanisms of Anthocyanins and Proanthocyanidins on Heterogeneous and Homogeneous Cation-Exchange Membranes

**DOI:** 10.3390/membranes11020136

**Published:** 2021-02-16

**Authors:** Véronique Perreault, Veronika Sarapulova, Ksenia Tsygurina, Natalia Pismenskaya, Laurent Bazinet

**Affiliations:** 1 Laboratoire de Transformation Alimentaire et Procédés Électromembranaires (LTAPEM, Laboratory of Food Processing and Electro-Membrane Processes), Food Science Department, Institute of Nutrition and Functional Foods (INAF), Université Laval, Québec, QC G1V 0A6, Canada; veronique.perreault.5@ulaval.ca; 2 Membrane Institute, Kuban State University, Stavropolskaya 149, 350040 Krasnodar, Russia; vsarapulova@gmail.com (V.S.); kseniya_alx@mail.ru (K.T.); n_pismen@mail.ru (N.P.)

**Keywords:** cation-exchange membranes, fouling, adsorption, desorption, proanthocyanidins, anthocyanins, cranberry juice, mechanisms

## Abstract

The presence of membrane fouling is the main drawback in membrane processes, and it is related to the premature use and high cost for the replacement of membranes. Polyphenols in cranberry juice are associated with ion-exchange membrane fouling, and it results in a loss of these beneficial compounds in the juice when treated by membrane processes such as electrodialysis. In the present work, four heterogeneous or pseudohomogeneous cation-exchange membranes (CSE-fg, MK-40, CEM Type-II, and CJMC-5), different in terms of the polymer matrix (aromatic, aliphatic), exchange capacity, size, and location of meso and macropores, were studied to understand the impact of the membrane structure and physico-chemical properties on adsorption and desorption of phenolic compounds (anthocyanins and proanthocyanidins) from cranberry juice. It appeared from these results that MK-40, CEM Type-II, and CSE-fg were more prone to fouling due to their high ion-exchange capacity, their thickness, and the presence of meso and macropores in their structure. Indeed, electrostatic interactions occurred between fixed groups of membranes and polyphenolic ions. Desorption of the entire membrane and cryogenic grinding with pH adjusted to 10 allowed a better recovery of anthocyanins and proanthocyanidins (PACs), respectively, since hydroxide ions competed with polyphenols and membrane that induced desorption of polyphenols. In the future, this new knowledge will become the basis for a more sensible choice of membranes and for the development of protocols for extending their life cycle.

## 1. Introduction

Membrane technologies are largely spread off in the food industry for producing, preserving, stabilizing, or separating food products. However, since food products are complex matrices, membrane fouling is a major issue in electrodialysis or, more generally, electromembrane process applications. It results in a loss of membrane permselectivity and an increase in membrane resistivity, which decreases the ion flux through the membrane and increases energy consumption. All these phenomena contribute to the shortening of membrane lifetime [1]. In addition, it has been reported that the cost for cleaning membranes and replacing them vary from 40% to 50% of the cost of the product [2,3,4], while the cost of membranes and spacers contributes to 25–30% of the total cost of a new electrodialysis unit fully automated [4]. Furthermore, cleaning in place procedures require the use of chemicals such as acid, bases, and sometimes surfactant or enzyme solutions that can lead to the swelling of the polymer matrix and further contribute to the adsorption of high molecular weight compounds [5]. Consequently, this can induce a premature renewal of membranes [5]. It is then necessary to understand the mechanisms of membrane fouling to prevent it and extend the lifetime of membranes.

There are several types of fouling that can be classified into different categories: Scaling, organic, colloidal, and biofouling. Among fouling linked with intrinsic compounds of a solution (scaling/minerals, organic/proteins, peptide, humic acid, colloidal/colloidal silica, clay minerals), colloidal and scaling fouling, as well as organic fouling by proteins and peptides, are now well documented, and mechanisms have been extensively studied to understand and avoid its formation on anion- and cation-exchange membranes [4,6,7,8,9,10]. However, it is not the case for organic fouling by polyphenols, which is present in several membrane applications such as tartaric stabilization of wine and deacidification of cranberry juice [11,12]. Indeed, wine and cranberry juice contain a large amount of polyphenols that can adsorb at the interface or inside the matrix of ion-exchange membranes. This adsorption does not only induce membrane fouling but also results in a loss of these compounds, having health benefits. Indeed, polyphenols are strong antioxidants having the potential to prevent oxidative damage due to reactive oxygen species and then protect against some cancers and cardiovascular diseases [13]. Furthermore, the consumption of cranberry juice is associated with a protection against urinary tract infections and against Helicobacter pylori infections [14]. Hence, it is important to understand from analytical and technological points of view the adsorption and desorption mechanisms of polyphenols on ion-exchange membranes to prevent membrane fouling and polyphenol loss.

Recent studies performed by Sarapulova et al. [15], Bdiri et al. [5], and [16] proposed mechanisms underlying the membrane fouling by polyphenols, but in these previous articles, only membranes with an aromatic polymer matrix were investigated. In addition, only the electrostatic mechanism of fouling was considered. Indeed, three main factors might influence the membrane fouling by polyphenols: (1) The type of polymer (aliphatic or aromatic), (2) the presence or absence of electrostatic interactions of polyphenols with fixed groups of the membrane, which depends on the exchange capacity of the membrane and on the external solution [17] and (3) the presence or absence of extended macropores [18], where no steric limitation to the transport of polyphenols exists and which transport largely depends on the adhesion of all membrane components to each other (reinforcing fabric or ion-exchange material). Consequently, there is still a lack of knowledge for polyphenol fouling, and to the best of our knowledge, no study has focused on the identification of the fouling by polyphenols in relation to the structure of heterogeneous and homogeneous cation-exchange membranes.

In this context, the goal of the present work was to study four heterogeneous or pseudo-homogeneous cation-exchange membranes with different characteristics to understand the impact of the membrane structure on the adsorption and desorption of phenolic compounds from cranberry juice. The specific objectives were to (1) characterize the cation-exchange membranes in terms of the thickness, conductivity, ion-exchange capacity, and volume fraction of the intergel space, (2) to evaluate the adsorption kinetic of polyphenols from cranberry juice on the membranes, (3) to evaluate two desorption methods based on membrane grinding or not and pH of the desorption and (4) to propose mechanisms explaining the effect of structure and chemical nature of the membranes on the adsorption and desorption of different PACs and anthocyanins present in cranberry juice.

## 2. Materials and Methods

### 2.1. Materials

#### 2.1.1. Cranberry Juice

Pasteurized and clarified cranberry juice was provided by Fruit d’Or (Plessisville, QC, Canada). The physicochemical characteristics of the juices are presented in Table 1.

#### 2.1.2. Membranes

Four cation-exchange membranes, with different characteristics, were used to evaluate polyphenol fouling (Table 2): CSE-fg (fg: Food grade, Astom, Tokyo, Japan), MK-40 (Shchekinoazot, Russia), Fuji CEM Type II (Fujifilm, The Netherlands), and CJMC-5 (Hefei Chemjoy Polymer Material Co. Ltd., Hefei, China). From many known ion-exchange membranes, 2 were chosen since they possessed an aromatic matrix (CSE-fg and MK-40) and 2 others were chosen for their aliphatic matrix (CEM Type-II and CJMC-5). In addition, 1 membrane has an aliphatic matrix and another one an aromatic matrix containing macropores in their structures (MK-40 and CJMC-5).

### 2.2. Methods

#### 2.2.1. Characterization of Pristine Membrane Properties

Dry membranes were pretreated by soaking in 300 g/L NaCl for 12 h and then in 30 g/L NaCl for 24 h. Thereafter, membranes were soaked in distilled water for another 24 h prior to use. Pretreated membranes were then soaked for 30 min in 500 mL of NaCl solution at a concentration of 0.1; 0.25; 0.50; 0.75, and 1.0 M to calculate the volume fraction of the intergel space. After this final soaking, membranes were characterized for their thickness and conductivity. The ion-exchange capacity of the pretreated membrane was also determined to complete the characterization of the initial membrane properties.

#### 2.2.2. Membrane Fouling Kinetics

**Figure 1:**
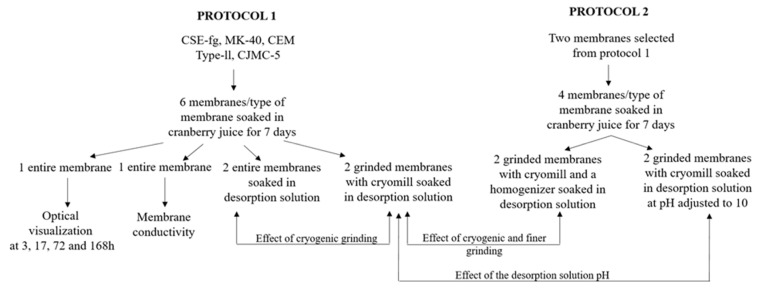
Design of the two desorption protocols.

**Figure 2:**
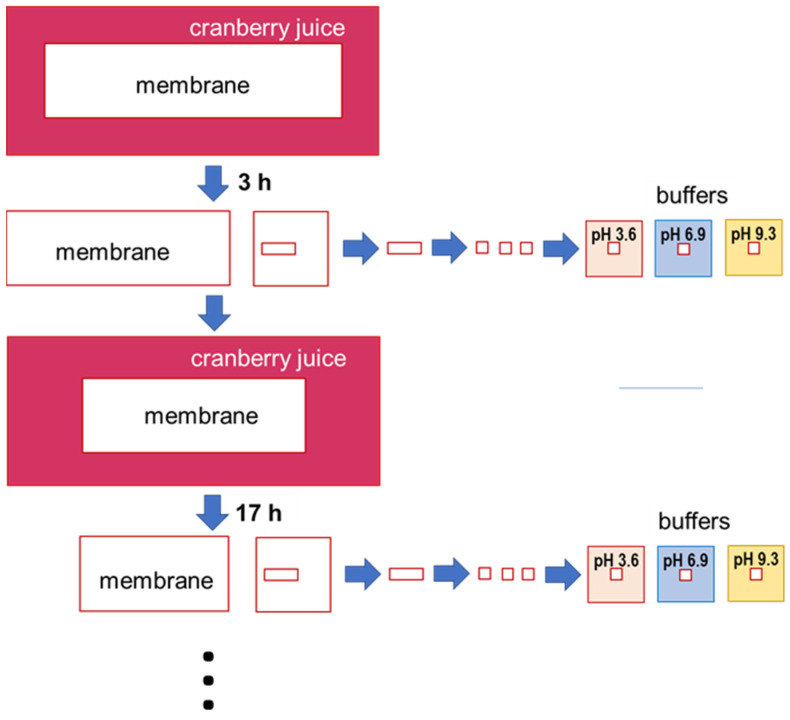
Coupon preparation for optical visualization of membrane fouling with cranberry juice.

For these experiments, 6 coupons of 10 cm^2^ for each membrane (CSE-fg, MK-40, CEM Type-II, and CJMC-5) were used (Figure 1-Protocol 1). They were soaked in cranberry juice without touching each other at a temperature of 10 °C. The juice was changed every 24 h during the 7 days (or 168 h) of soaking to induce PAC and anthocyanin fouling. Among these 6 coupons:

One coupon was used for the fouling kinetic study by optical microscopy (Figure 1-Protocol 1). Hence, 2.5 cm^2^ was cut from the long side of this coupon after 3, 17, 72, and 168 h of soaking in cranberry juice (Figure 2). From each 2.5 cm^2^ coupon, 3 coupons (one for each pH) of 3 × 3 mm^2^ of membranes were cut and placed in a buffer solution at pH of 3.6, 6.9, and 9.3 for 2 h (Figure 2). Weakly acidic, neutral, and slightly alkaline pH values were chosen to demonstrate more clearly the sorption of anthocyanins, which color changed depending on the solution pH [20]. Optical images of the surface and cross-section of pristine and fouled coupons were then obtained by optical microscopy. In parallel, optical images of the cranberry juice at different pH values were also obtained to produce a color scale as previously reported by Pismenskaya et al. [21]. The juice was diluted 10 times before recording color. Its pH was corrected by adding 1M HCl or NaOH solutions.

One coupon, after soaking for 168 h in cranberry juice, was used for conductivity measurements (Figure 1-Protocol 1). It was placed in 200 mL of 1M NaCl solution for 1 h (Figure 1-Protocol 1). Then, the solution was replaced by a fresh 200 mL of 1M NaCl solution, and the electrical conductivity of the membrane was measured.

The 4 other coupons were used for the 1st desorption procedure (Figure 1-Protocol 1) concerning the effect of grinding (see Section 2.2.3.1).

#### 2.2.3. Desorption Procedures

Two desorption protocols (Figure 1) were tested optimize PAC and anthocyanin desorption from fouled membranes based on the previous method developed by Bdiri et al. [5]. These 2 protocols allowed the evaluation of 3 different effects: Cryogenic grinding (Cryomill), cryogenic grinding followed by a further finer grinding with a homogenizer (Ultra-Turrax), and the pH of the desorption solution on PAC and anthocyanin content (pH 6 vs. pH 10).

##### 2.2.3.1. Protocol 1: Effect of Grinding on Desorption

Two coupons were used for interfacial desorption and 2 others for cryogenic grinding before desorption (Figure 1-Protocol 1). The 1st 2 coupons were soaked in 20 mL of a mix of a solvent composed of 25% acetonitrile/25% methanol/25% isopropanol/25% water [5] during 3 h at 10 °C under stirring to desorb PACs and anthocyanins at the interface of the membranes. The 2 other coupons were cut into small pieces and ground at 25 Hz for 2 min (Cryomill, Retsch, Germany), as seen in Bdiri et al. [5], before desorption in 20 mL, also for 3 h. The solutions were then centrifuged at 5000× *g* for 5 min at 4 °C to remove the membranes, and the supernatant was collected. For both desorption methods, 10 mL were collected for PACs analyses and the last 10 mL for anthocyanins analyses. The solutions were vacuum dried (Savant SPD131DDA SpeedVac concentrator, Thermo Scientific, MS, USA) and then reconstituted in the analysis solutions of PACs and anthocyanins.

##### 2.2.3.2. Protocol 2: Effect of Finer Grinding and pH of the Desorption Solution on Desorption

For this 2nd protocol aiming at the optimization of the desorption, based on the results of the 1st protocol, the membranes presenting the highest concentrations of PACs and anthocyanins, and the membrane presenting intermediary results among the 3 other types of membranes were selected. For each repetition, 2 of the 4 coupons were used for cryogenic grinding desorption and the 2 others for cryogenic grinding followed by a homogenizer (Ultra-Turrax, Ika, Wilmington, NC, USA) before desorption to achieve a finer grinding to potentially increase polyphenols desorption (Figure 1-Protocol 2). The 1st 2 ground coupons (25 Hz, 2 min, Cryomill, Retsch, Germany) were soaked in 20 mL of a mix of a solvent composed of 25% acetonitrile/25% methanol/25% isopropanol/25% water adjusted at pH 10 [5] during 3 h at 10 °C under stirring to desorb PACs and anthocyanins from the membranes. The 2 other ground coupons (25 Hz, 2 min, Cryomill, Retsch, Germany) were soaked in 20 mL of desorption solution without adjusted pH (pH 6) before being homogenized again with Ultra-Turrax for 1 min to achieve a finer grinding. Samples were then stirred during 3 h at 10 °C. Afterward, the solutions were centrifuged at 5000 g for 5 min at 4 °C to remove the membranes, and the supernatant was collected. As previously, for both desorption methods, 10 mL were collected for PACs and anthocyanin analyses and, the solutions vacuum dried to be reconstituted in the analysis solutions of PACs and anthocyanins.

#### 2.2.4. Analyses

##### 2.2.4.1. Optical Microscopy

Optical images of surface and cross-section of pristine and fouled swollen coupons were obtained using an optical microscope SOPTOP (model CX40M, NINGBO SUNNY INSTRUMENT CO. LTD, China) with a magnitude of 10X (surface) and 5X (cross-sections), in transmitted light mode. Optical images of the cranberry juice at different pH values were obtained at constant luminous intensity (ensured by the constant electric power 14 W ± 5% consumed by the light source) and with an optical path length of 225 mm.

##### 2.2.4.2. Thickness

Membrane thickness was measured with a Marathon Electronic Digital Micrometer (Marathon Watch Compagny Ltd., Richmond Hill, ON, Canada). Six measurements at different positions on the membrane were recorded and averaged. 

##### 2.2.4.3. Ion-Exchange Capacity

Ion-exchange capacity (IEC) corresponded to the number of active sites and was measured according to Bazinet et al. [22]. Briefly, membranes were soaked overnight in 1N HCl, rinsed with distilled water, and soaked again in a known volume of 0.1 N NaOH for 15 min. The membrane was then rinsed with distilled water. The rinsed water was mixed with the NaOH solution and titrated with 0.5 N HCl. The IEC was calculated according to Equation (1).

(1) $$IEC=\frac{\left(V_{NaOH}\times \left[NaOH\right]\right)-\left(V_{HCl}\times \left[HCl\right]\right)}{Mm}$$

where IEC was the number of mmol per gram of wet membrane, V_NaOH_ and [NaOH] the volume (mL) and the concentration of NaOH solution (N), V_HCl_ and [HCl] the volume (mL), and the concentration of the HCl solution (N) used for the titration of the NaOH solution; Mm was the mass of the dry membrane.

##### 2.2.4.4. Conductivity

Membrane conductivity was measured with a specifically designed clip from the Laboratoire des Matériaux échangeurs d’Ions (Université Paris Xll, Créteil, France) with 1 cm between the electrodes and a 0.5M NaCl reference solution. The membrane conductivity was calculated according to Lteif et al. [23] and Lebrun et al. [24].

##### 2.2.4.5. Volume Fraction of the Intergel Space

The volume fraction of the intergel space, f2, was localized in the central part of meso-, macropores, and structural defects of the membranes. It is filled with an electrically neutral solution, which was identical to the external solution. The remaining membrane volume (f1 = 1 − f2) filled the gel phase. This phase included a polymer matrix with fixed charged groups, as well as a charged solution of mobile counter-ions (and the co-ions in a smaller amount), which compensated the charge of the fixed groups. The reinforcing cloth or fibers, as well as inert filler, were considered as parts of the polymer matrix [25]. Then, the volume fraction of the intergel space (f2) was determined from the membrane conductivity measured at different NaCl concentrations. Indeed, according to the microheterogeneous model described by Zabolotsky and Nikonenko [26], the slope of these dependencies presented in Log-Log coordinates gave the value of the volume fraction of intergel space, f2.

##### 2.2.4.6. Electrical Conductivity and Ion-Exchange Capacity of the Gel Phase

The volume fraction of the intergel space allows the estimation of the electrical conductivity of the gel phase, $\bar{\kappa }$ (in mS/cm), according to the following equation [26]:

(2) $$\mathrm{Log}\;(\bar{\kappa })=b/f1=b/(1-f2)$$

where b, is the free term in the equation describing the linear dependencies in Log-Log coordinates of the membrane conductivity measured as a function of the conductivity of the NaCl solution.

The volume fraction of the intergel space also leads to the determination of the ion-exchange capacity of the gel phase according to Equation (3) [26]:

(3) $$\bar{Q}=k/(1-f2)$$

##### 2.2.4.7. Proanthocyanidin Content

For PAC analysis, samples were solubilized in 1 mL of a solution of acetone/water/acetic acid (79.9/20/0.1) prior to filtration through a 0.45 µm nylon filter. Samples were analyzed according to Faucher et al. [27] onto an Agilent 1260 series HPLC system with a fluorescence detector to measure the PAC profile on a Nomura chemical Develosil 100 Diol-5 (250 × 4.6 mm, Phenomenex, Torrance, CA, USA) column at 35 °C. A volume of 5 µL of samples was injected. PACs were separated with 2 solvents at a flow rate of 0.8 mL/min: Solvent A, acetonitrile/acetic acid (98%/2%), and solvent B, methanol/water/acetic acid (95%/3%/2%). Emission and excitation wavelength were set at 321 nm and 230 nm, respectively. To quantify the PACs of different degrees of polymerization, a calibration curve of epicatechin was used and a correction factor to convert the different response factors of monomeric to polymeric PACs. Each PAC content was expressed as mg/L epicatechin equivalents. Samples were concentrated 10 times, thus PAC content was divided by ten, which corresponded to the concentration of the initial desorption solution. Since the volume of samples differed from each other, for comparison purposes, the PACs contents were divided by the volume of the membrane. Results were presented in μg of PACs/cm^3^ of the membrane. Calculations were done according to the thickness of the membranes soaked in NaCl 0.1 M since this solution had the closest conductivity closest to the cranberry juice, among all tested conductivities.

##### 2.2.4.8. Anthocyanin Content

For anthocyanin analysis, samples were solubilized in 1 mL of a solution of water/methanol/TFA (70/29.5/0.5) prior to filtration through 0.45 µm nylon filter for both analyses. According to Faucher et al. [27], individual anthocyanin composition was analyzed by HPLC using an Agilent 1100 series system equipped with a diode array detector. A volume of 20 µL of samples was injected and analyzed with a Zorbax SB-C18, 5 µm (250 × 4.6 mm, Agilent, Santa Clara, CA, USA) column at room temperature. Two solvents were used for elution at 1 mL/min: Solvent A, water/formic acid (95%/5%), and solvent B, methanol (100%). The detection wavelength was set at 520 nm. Anthocyanins content were expressed in mg/L. Samples were concentrated 10 times, thus PAC content was divided by 10, which corresponded to the concentration of the initial desorption solution. As previously for PACs, results were presented in μg of anthocyanins/cm^3^ of membrane, based on the membrane thickness in NaCl 0.1 M.

### 2.3. Statistical Analyses

Statistical analyses were performed using Sigma Plot software (version 14, Systat, San Jose, CA, USA). Data were reported as mean value ± standard deviation. One-way ANOVA was conducted to determine statistical differences between membrane conductivities and NaCl concentrations with a Tukey test, at a confidence level of 0.05. A *t*-test was also conducted at a confidence level of 0.05 for PACs and anthocyanins analyses.

## 3. Results and Discussion

### 3.1. Characterization of the Pristine Membranes

#### 3.1.1. Optical Microscopy

**Figure 3:**
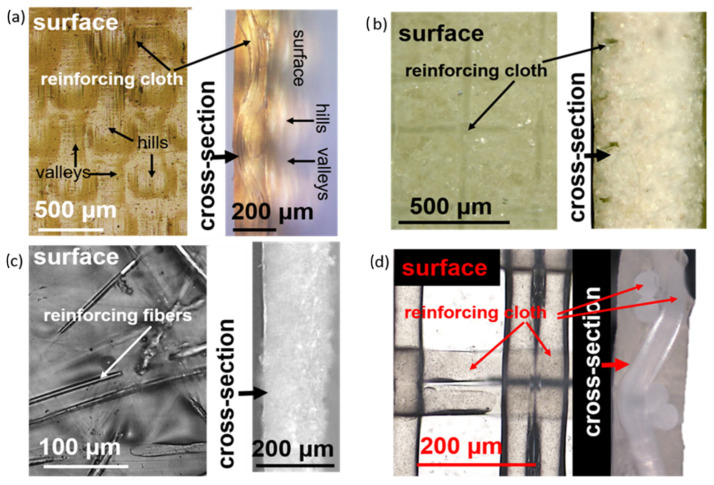
Optical images of the surface and cross-sections of pristine membranes CSE-fg (**a**), MK-40 (**b**), CEM Type-II (**c**), and CJMC-5 (**d**) in a 1.0 M NaCl solution.

Optical images of the surface and cross-sections of coupons of CSE-fg, MK-40, CEM Type-II, and CJMC-5, membranes in a 1.0 M NaCl solution showed the location of the reinforcing cloths of the pristine membranes (Figure 3).

It appeared that the reinforcing cloth penetrated the entire CJMC-5 and CSE-fg cross-sections. For this last membrane (CSE-fg), the reinforcing cloth embedded in the membrane provided a wavy surface with “hills” and “valleys.” The structure of this new membrane was very similar to that of the well-studied AMX and CMX membranes from the same manufacturer [15,28,29]. In the case of MK-40, two reinforcing clothes were located near the membrane surfaces while the reinforcing cloth of the CEM Type-II membrane randomly penetrated its entire volume. Some of the CEM Type-II fibers coated with ion-exchange material came close enough to the surface. The space between the fibers was filled with homogeneous ion-exchange material.

#### 3.1.2. Thickness

Globally, for the same type of membrane, its thickness (Table 3) was quite similar, whatever the NaCl concentration, indicating that membrane swelling did not change. This was in accordance with Dlugolecki et al. [30], who reported that the swelling degree of Neosepta CMX membranes (a variant of CSE) was independent of the NaCl concentration since the cross-linking degree and reinforcement gave membranes high mechanical stability. In addition, it is important to mention that the thickness of MK-40 was 3 times the thickness of the other investigated membranes: ~523 μm vs. 174, 150, and 140 μm for CEM Type-II, CJMC-5, and CSE-fg, respectively.

#### 3.1.3. Ion-Exchange Capacity

IEC of membranes in a swollen state, determined according to a titration as described in Section 2.2.4.3, was used to describe the extent of fixed charged group functionalization. CSE-fg presented the highest IEC of 1.61 ± 0.05 mmol/g (*p* < 0.05), while MK-40 and CEM Type-II presented a similar IEC of 1.43 ± 0.08 and 1.35 ± 0.05 mmol/g, respectively (*p* < 0.05). CJMC-5 had the lowest IEC of the four membranes with a value of 0.57 ± 0.05 mmol/g (*p* < 0.05). The fact that CSE-fg and MK-40 had the highest IEC indicated that they contained more fixed charged groups into their polymer matrix [31].

#### 3.1.4. Conductivity

**Figure 4:**
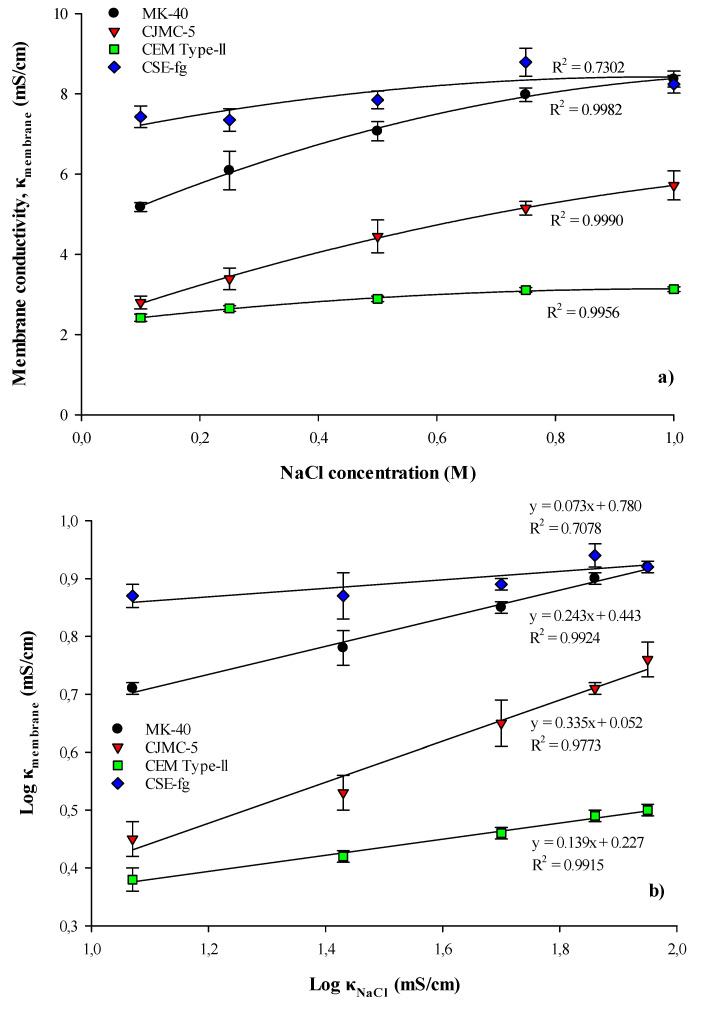
Concentration dependencies of (**a**) specific electric conductivity of the studied membranes, κ_membrane_ (mS/cm), and (**b**) the same dependencies presented in logarithmic coordinates.

Figure 4a demonstrated that for all membranes, the conductivity increased with the NaCl concentration. Since the conductivity of cranberry juice was 3817 µS/cm, the membrane conductivities measured in 0.1M NaCl were the closest to cranberry juice. Globally, for all NaCl concentrations, CSE-fg had the highest conductivity, followed by MK-40, CJMC-5, and CEM Type-II (*p* < 0.05). Furthermore, the conductivity increased sharply for MK-40 and CJMC-5 with NaCl concentration. These results were in accordance with the IEC values and confirmed that the higher conductivity of CSE-fg and MK-40 was linked to the higher number of fixed charged groups (SO_3_H). This was also in accordance with the results obtained by Gohil et al. [32] with homogeneous, heterogeneous, and interpolymer cation and anion-exchange membranes who supported the increase in conductivity with the IEC values.

The log κ of membranes according to the log of NaCl concentration in solutions was presented in Figure 4b. It appeared from these results that f2, the volume fraction of the intergel space [26], were significantly different for all membranes (*p* < 0.05). CJMC-5 and MK-40 membranes had the highest volume fractions of the intergel space, with values of 34% and 24%, respectively, compared to CEM Type-II and CSE-fg (13.9 and 7.3%, respectively) (Table 4). As expected, the gel phase-specific electrical conductivity values (in mS/cm), increased in the following sequence: CJMC-5 < Fuji CEM Type-II < MK-40 < CSE-fg (Table 4), that was, in the same sequence in which the exchange capacity of the membranes increased (see Section 3.1.3). However, the ion-exchange capacity of the gel phase increased in the following sequence: CJMC-5 < CEM Type-II < CSE-fg < MK-40, with small differences between CSE-fg and MK-40 (Table 4).

It was known that the point of isoelectric conductivity, where the electrical conductivity of the gel phase was equal to the electrical conductivity of the membrane, was in the region of the centimolar NaCl solutions [33]. However, in more concentrated solutions, such as in our experiments (Figure 4a), the electrical conductivity of the membranes was mainly determined by the electrical conductivity of the solution filling the intergel space. The contribution of this electrical conductivity to the total electrical conductivity of the membranes increased with the increasing concentration of the external solution. In the case of MK-40 and CEM Type-II, these data were in good agreement with the results presented in Sarapulova et al. [34], who reported conductivities (κ) of 2.8 ± 0.11 mS/cm and a variation from 5.27 to 8.3 mS/cm for CEM Type-II and MK-40, respectively. Since the CJMC-5 and CSE-fg membranes are relatively new, this was probably why their specific conductivities were not found in the scientific literature. However, the concentration dependencies of the electrical conductivity of CSE-fg membrane were close to the dependencies obtained previously for the CMX membrane from the same manufacturer.

To link membrane structures and f2 values, previous studies reported that membranes with f2 > 0.2 (such values are mainly characteristic of heterogeneous membranes, in particular, MK-40 membranes) have large macropores with effective radii of about 150 nm and 3000 nm [15,34,35,36]. The first was formed between the particles of the ion-exchange resin and an inert filler. The second was localized around the threads (fibers) of the reinforcing cloth. Some of these pores came to the surface [37], which facilitated the diffusion delivery of high molecular weight substances into the membrane volume [34]. CJMC membranes were specially designed to allow the transfer of large, including organic, ions [38]. Apparently, the combination of large pores of the CJMC-5 ion-exchange material with extended pores around the threads of the reinforcing fabric was the reason for its maximum f2 value among the studied membranes. In contrast, in membranes for which f2≈0.1 (CSE-fg and CEM Type-II), mesopores with effective radii that did not exceed 15 nm dominate. In addition, there was a small number of structural defects with an effective pore radius of not more than 40 nm (macropores) [15,34,35,36].

### 3.2. Membrane Fouling Kinetics

#### 3.2.1. Membrane Conductivity after Soaking in Cranberry Juice

**Figure 5:**
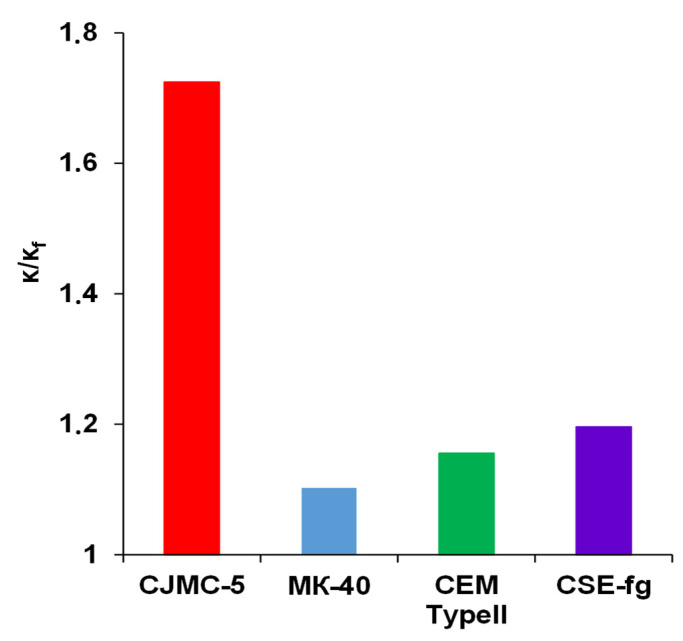
Ratios of the specific electrical conductivities of pristine membranes and membranes soaked in cranberry juice for 168 h.

The ratio of the specific electrical conductivities of CJMC-5 pristine membrane and membrane in contact with cranberry juice after 168 h decreased most noticeably (1.7 times) compared to pristine membranes (Figure 5). In the case of MK-40, CEM Type-II, and CSE-fg fouled membranes, the electrical conductivity decreased only by 1.1–1.2 times. Since the electrical conductivity of a membrane was related to its charges (or number of fixed groups), the decrease in conductivity compared to the pristine ones was mainly due to the electrostatic interactions of their fixed groups with polyphenol cations [16]. The more the proportion of fixed groups that have entered into such interactions, the more noticeable was the relative decrease in electrical conductivity and, correspondingly, an increase in the electrical resistance of membranes. This was in accordance with previous results of IEC (see Section 3.1.3). Indeed, since the CJMC-5 had the lower ion-exchange capacity of the pristine membranes, it was more affected by the polyphenol cations, which resulted in a reduced electrical conductivity. This was also why the electrical resistance of CJMC-5 membrane was the most sensitive to polyphenol poisoning compared to other membranes studied. Pristine membranes MK-40, CEM Type-II, and CSE-fg had close exchange capacity values. However, the electrical conductivity of the thickest MK-40 membrane was reduced the least after soaking in cranberry juice. It can be hypothesized that the main reasons for the low reduction of electrical conductivity of MK-40 were (1) the length of the path that polyphenols must cover to fill the entire volume of the membrane, as well as (2) the presence of extended macropores that reach the surface of the membrane and facilitate the penetration of anthocyanins deep into its volume. Indeed, several studies showed that heterogeneous membranes MA-41 and MK-40 contained such macropores [34,36,37] and that these extended macropores were localized at the boundaries of the ion-exchange resin particle/inert binder and at the boundaries of the reinforcing fabric thread/ion-exchange composite.

#### 3.2.2. Fouling Kinetics and Color Indication of Anthocyanins

**Figure 6:**
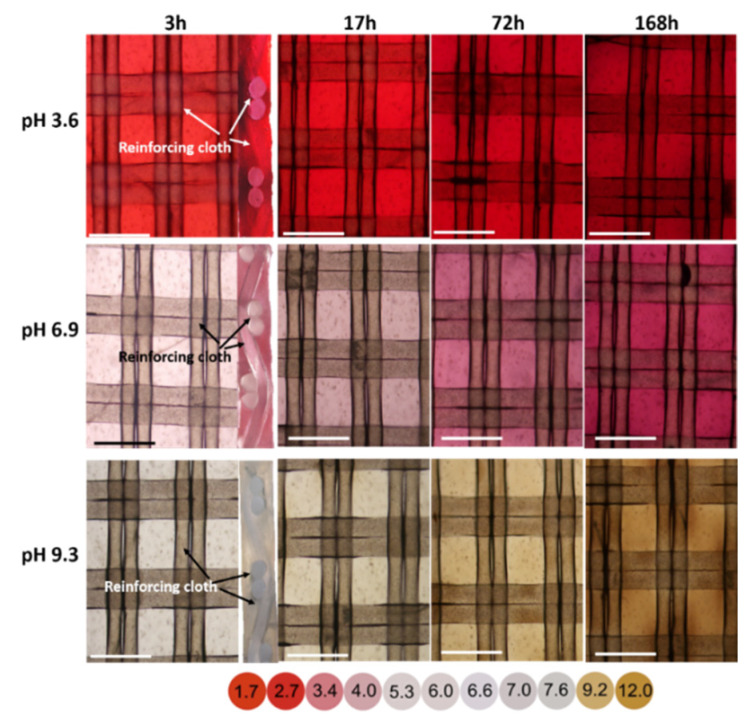
Optical images of the surfaces (left) and sections (right) of CJMC-5 membrane coupons that were in contact with cranberry juice for 3, 17, 72, and 168 h. Before obtaining images, these coupons were placed for 2 h in buffer solutions at pH 3.6, 6.9, and 9.3. The dependence of cranberry juice pH (diluted 10 times) on its color is shown below the figure. The scale is 200 μm.

Optical images of the surface and cross-sections of CJMC-5 membrane coupons that were in contact with cranberry juice for 3, 17, 72, and 168 h were presented in Figure 6. Before obtaining such images, these coupons were placed for 2 h in buffer solutions at pH of 3.6, 6.9, or 9.3 and were compared in terms of color with a color scale. This color scale showing the evolution of cranberry juice (diluted 10 times) as a function of pH is presented in Figure 6. In addition, Optical images showed that the color of the membrane changed from red (acidic pH) to a brownish color (alkaline pH).

The color of cranberry juice observed at pH < 3, was characteristic of cyanidins [39], the concentration of which was dominant in the composition of anthocyanins (Table 1). Indeed, blueness was enhanced by increasing free hydroxyl groups, whereas redness intensified with the raising of the methylation of the hydroxyl groups as indicated by peak wavelengths in [39]. The identified anthocyanins, considering their ability to change color depending on the pH, filled the entire volume of the membrane already after 3 h of soaking in cranberry juice (Figure 6). Judging by the color of the cross sections of the coupons, the pH of the internal solution of the membrane was 1.5–2 units lower compared to the external solution (Table 5). This experimental fact was due to Donnan’s exclusion of protons from the membrane, which were the products of the photolysis reactions of anthocyanins and other components of cranberry juice. A similar phenomenon in the case of ion-exchange resins, which were soaked in a model solution of anthocyanins, was observed in Sarapulova et al. [15] and [21]. The CJMC-5 membrane had rather large macropores in the ion-exchange material in addition to the macropores localized in the threads of the reinforcing fabric. Therefore, the entire volume of this membrane was fouled with anthocyanins. This explained why this membrane lost its electrical conductivity to a greater extent than other studied membranes. Hence, with the presence of macropores, faster fouling by anthocyanins occurred with the CJMC-5, and easier anthocyanins desorption was expected. Therefore, these substances quickly occupied the entire membrane volume. However, the size of these pores was not so large to facilitate the diffusion of PACs and other large molecules. Note that the presence of macropores, which was the reason for the faster fouling of the CJMC-5 with anthocyanins, gave hope for easier extraction of anthocyanins from this membrane.

**Figure 7:**
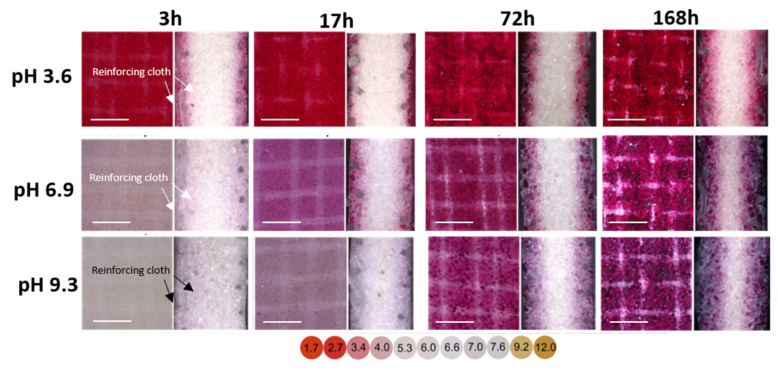
Optical images of the surfacesthe total concentration of anthocyanins and PACs in these solutions increased in the same sequence (left) and cross-sections (right) of MK-40 membrane coupons that were in contact with cranberry juice for 3, 17, 72, and 168 h. Before obtaining images, these coupons were placed for 2 h in a buffer solution at pH 3.6, 6.9, and 9.3. The dependence of cranberry juice pH (diluted 10 times) on its color is shown below the figure. The scale is 300 μm.

Concerning MK-40 membranes, it appeared that two reinforcing clothes were located near the membrane surfaces (Figure 7). Furthermore, in the first 3 h of soaking, anthocyanins appeared in the ion-exchange composite (a mixture of particles of ion-exchange resin with an inert binder—polyethylene), which was adjacent to the reinforcing cloth. An increase in the soaking duration of MK-40 in cranberry juice gradually decreased the thickness of the anthocyanin free-layer located in the middle cross-sections of coupons (Figure 7, Table 6). However, the mid-section of the membrane remained free of anthocyanins after 168 h of soaking. The color intensity of the MK-40 surface and near-surface layers increased from the prolonged soaking of the membrane in cranberry juice (Figure 7). Moreover, this color acquired a red-brown-blue shade as the soaking duration was extended. This shade was characteristic of the associations of anthocyanins with tannins and other components of juices [40]. Apparently, relatively small molecules [39] of free (individual) anthocyanins entered the MK-40 membranes, primarily due to diffusion in the macropores, which were localized in the threads of the reinforcing cloth. Then they spread to the nearby space due to the pores between the resin particles and the inert binder. The increase in the thickness of the MK-40 membrane was also in favor of the presence of a stretching phenomenon (Table 6).

Indeed, strongly hydrated carboxylic acids and amino acids, which entered the internal solution of the membranes from juice, were the cause of the stretching of the ion-exchange matrix. This phenomenon was observed for ion-exchange membranes soaked in a solution of hydrotartrate [41]. As a result, the pores of the ion-exchange polymer were enlarged. Therefore, relatively small molecules of anthocyanins more easily entered the ion-exchange material. As a result, large aggregates or polymers of anthocyanins with each other and with other components of the juice penetrated the ion exchange membrane. However, the inner part of the thick MK-40 membrane remained free of anthocyanins and their aggregates with other substances. Therefore, its electrical conductivity decreased less than would be expected.

**Figure 8:**
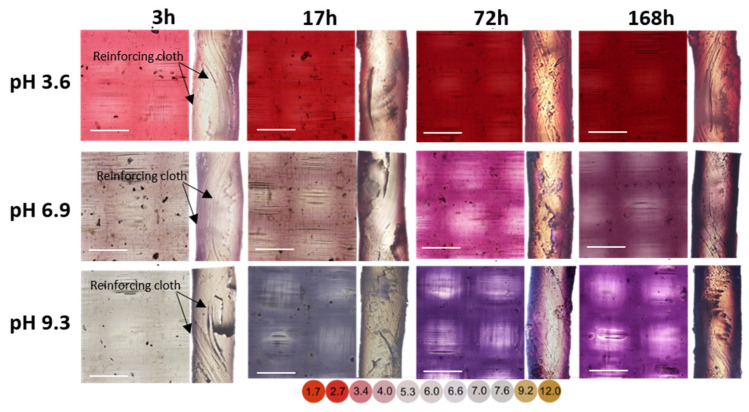
Optical images of the surface (left) and cross-sections (right) of CSE-fg membrane coupons that were in contact with cranberry juice for 3, 17, 72, and 168 h. Before obtaining images, these coupons were placed for 2 h in a buffer solution at pH 3.6, 6.9, and 9.3. The dependence of cranberry juice pH (diluted 10 times) on its color is shown below the figure. The scale is 300 μm for membrane surface and 150 µm for cross-section.

The kinetics of fouling of CSE-fg coupons differs from that observed in the case of CJMC-5 and MK-40 membranes. According to the values of f2 (Figure 4b and Table 4), CSE-fg membrane had practically no macropores. Indeed, anthocyanins and other colored substances did not penetrate its volume through the reinforcing cloth/ion exchange composite boundaries and other structural defects (Figure 8). The front of these substances moved from the surfaces into the depth of the membrane. Moreover, with an increase in the soaking duration in cranberry juice, two layers became more and more visible. The darker layers of foulants (apparently anthocyanins, mainly) took up almost the entire volume of the coupons after 168 h, leaving only a small gap in the middle of the cross-section of the membrane. This darker blue-brown layer was mainly localized on the surface of the coupons but gradually penetrated inside CSE-fg and occupied up to 40% of the thickness of the cross-section of this thin membrane. Thus, after a given contact time with cranberry juice, the proportion of the CSE-fg volume poisoned by polyphenols was higher compared to the thicker MK-40 membrane. This was the reason for the more significant loss of CSE-fg electrical conductivity in comparison with the MK-40 membrane (Figure 5). CEM Type II membrane exhibited intermediate fouling kinetic (not shown) and, accordingly, loss of electrical conductivity (Figure 5) between CSE-fg and MK-40. These results of fouling kinetics and color indication of anthocyanins confirmed the previous hypothesis proposed in Section 3.2.1.

### 3.3. Desorption of Polyphenols from Membranes

To assess the relationship between the characteristics of the membrane and the degree of fouling with polyphenols, membrane coupons were soaked in a mixture of aliphatic solvents (25% acetonitrile/25% methanol/25% isopropanol/25% water) containing polar (including hydroxyl) groups. These solvents were targeted to dissolve hydrophilic polar substances due to electrostatic interactions and the formation of strong hydrogen bonds. It was expected that such a mix of solvent (pH 6) would have a greater effect on polyphenols, which were retained in the membranes due to electrostatic interactions and hydrogen bonds. In addition, and to a smaller extent, it will desorb polyphenols, which were adsorbed by the membrane due to π-π (stacking) interactions.

#### 3.3.1. Entire Coupons

**Figure 9:**
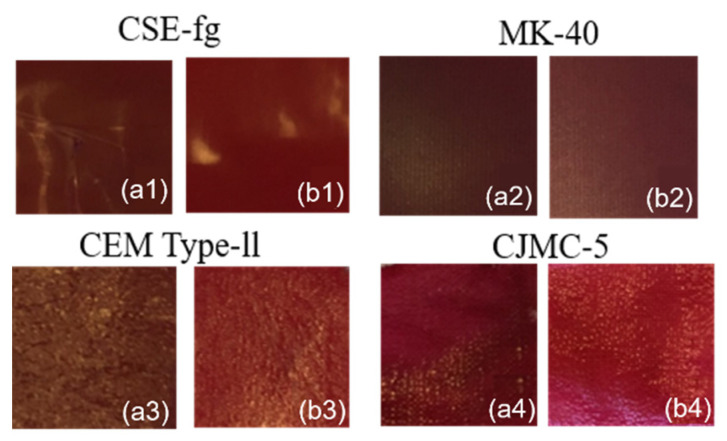
Photos of entire membranes before (**a1**–**a4**) and after (**b1**–**b4**) treatment in 25% acetonitrile/25% methanol/25% isopropanol/25% water at pH 6.

**Figure 10:**
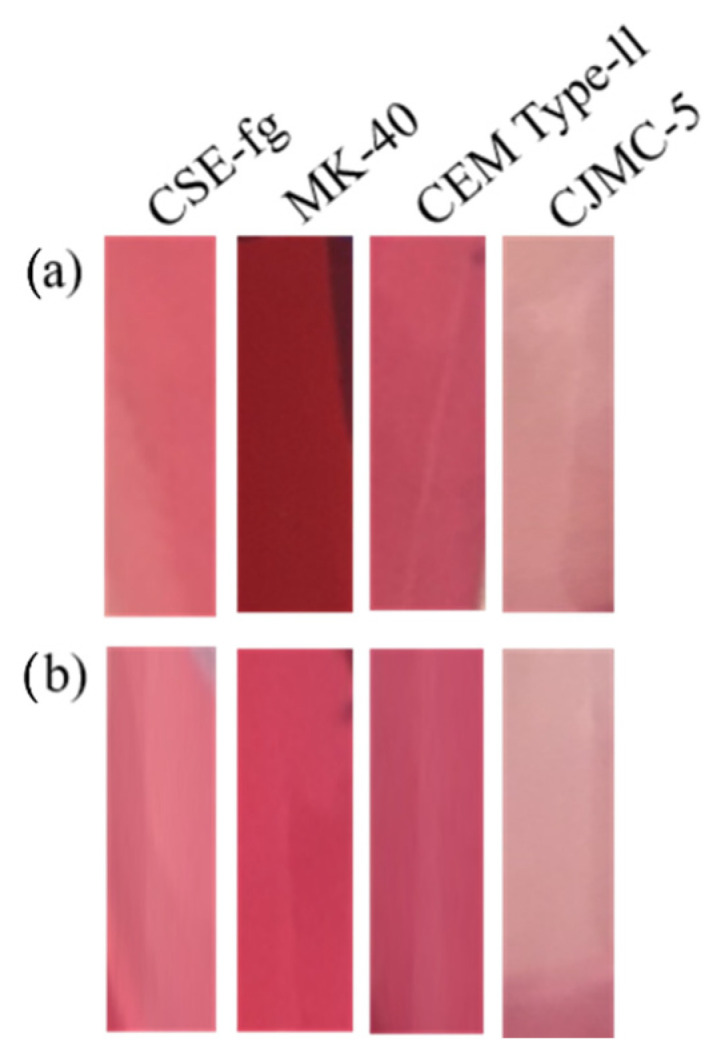
Photos of desorption solutions after the treatment of (**a**) entire coupons and (**b**) ground (cryogenic grinding) coupons.

The desorption treatment with 25% acetonitrile/25% methanol/25% isopropanol/25% water at pH 6 (Protocol 1) of the entire membranes resulted in lightening and reddening of the surface of the aliphatic membranes. At the same time, the brown color of the surface of aromatic membranes changed slightly whatever the membrane type (Figure 9). Such an impact of the desorption treatment was confirmed by the color intensity of the solution after the desorption stage, which increased in the series CJMC-5 < CSE-fg < CEM Type-II << MK-40 (Figure 10).

In addition, the total concentration of anthocyanins and PACs in these solutions increased in the same sequence (Table 7 and Table 8). All types of PACs from monomers to 4mers were only detected for the entire MK-40 coupon. At the same time, the red-brown-blue shade of the surface and sub-surface layers of the CSE-fg coupon after prolonged contact with cranberry juice (Figure 9 and Figure 10) was an indicator of the presence of these substances. All anthocyanins contained in cranberry juice were found only in the case of the MK-40 membrane. However, their concentration turned out to be lower than after soaking CEM Type-II membrane in a mixture of solvents. The smallest amount of anthocyanins was detected in the lightest solution obtained after soaking the CJMC-5 membrane. However, the concentration of anthocyanin detected was very low, comparable to the error of the determination method, which probably indicated that the fouling by anthocyanins was very limited. In addition, cyanidin-3-glucoside and peonidin-3-glucoside, which concentrations in cranberry juice were the lowest, were absent in the desorption solution of CEM Type-II, CJMC-5, and CSE-fg membranes. In general, anthocyanins concentrations in the solution increase in the series CJMC-5 < CSE-fg < MK-40 < CEM Type II.

**Figure 11:**
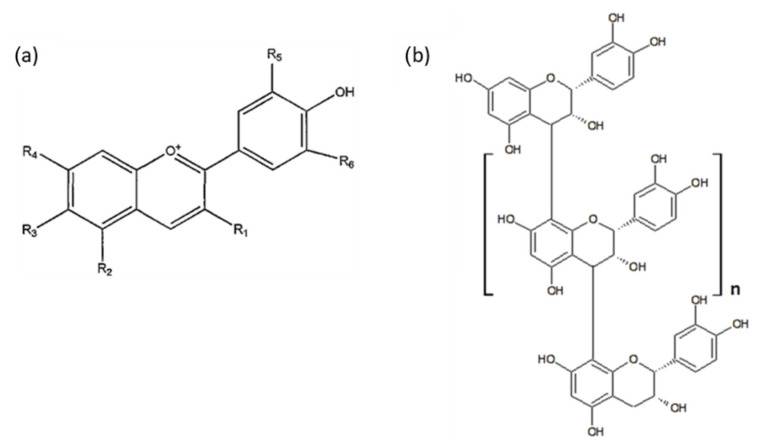
Schematic structure of individual anthocyanins (**a**) and proanthocyanidins (PACs) (**b**) [42,43].

Thus, as expected, part of the polyphenols was not desorbed and remained on the surface as well as inside MK-40 and CSE-fg membranes. This was attributed to the fact that π-π (stacking) interactions between anthocyanins and the aromatic matrix in some way counteracted the extraction of these polyphenols from MK-40 and CSE-fg if polar aliphatic desorption solutions were used. The highest adsorption of anthocyanins due to electrostatic interactions with fixed groups in comparison with other membranes was supported by the highest exchange capacity of the MK-40 gel phase (Table 4). Treatment with the desorption solution led to the destruction of these interactions and hydrogen bonds. In addition, the presence of large macropores in the structure of the MK-40 membrane facilitated the anthocyanin desorption. It is important to mention the facility of desorption of the anthocyanins versus the PACS since PACs molecular weight and size are several times greater than that of anthocyanins (Figure 11). Such large substances can adsorb on the surface or be located in membranes macropores. Almost 80% of the MK-40 membrane surface was covered with polyethylene, on which PACs can be retained due to hydrogen bonds or hydrophobic interactions. Extraction of PACs from this part of the surface and from macropores provided the highest total concentration of polyphenols in the MK-40 desorption solution. The ion-exchange capacity of CSE-fg gel phase (Table 4) was comparable to MK-40 and had predetermined the high concentration of anthocyanins in this membrane due to electrostatic interactions. However, the absence of macropores in the CSE-fg membrane, which caused steric hindrances, limited anthocyanins, and PACs diffusion deep into the CSE-fg membrane and back during desorption. Thus, the narrow pores of this membrane prevented the desorption of PACs and anthocyanins under the experimental conditions. Otherwise, it can be hypothesized that molecules such as polyphenols can enter the pores of the membrane, but they could not be released in the desorption solution due to the chemical nature of the solvents or pH of the solution.

CEM Type-II and CJMC-5 aliphatic membranes did not enter π-π (stacking) interactions with anthocyanins and PACs. This was why these membranes adsorbed less polyphenols and especially PACs, which had no electrical charge. At the same time, the amount of anthocyanins adsorbed due to electrostatic interactions increased in the same sequence as the ion exchange capacity of the gel phase: CJMC-5 < CEM Type-II (Table 4). Therefore, CJMC-5 contribution to the adsorption of anthocyanins due to electrostatic interactions was minimal. Even though CEM Type-II membrane had fewer macropores compared to the CJMC-5 membrane, the number of macropores seemed to be enough to provide desorption of a significant amount of anthocyanins during CEM Type-II membrane treatment with a mixture of solvents. As a result, a lower quantity of anthocyanins was desorbed from the CJMC-5 membrane, which had a higher number of macropores, but the lowest gel phase ion-exchange capacity (Table 4).

It is important to mention at this step that the breakage of electrostatic interactions was achieved only if the anthocyanins, in cationic forms in acidic cranberry juice, became neutral molecules (no interaction with the fixed groups of the membrane) or turned into anions (the charge coincides with the charge of fixed groups and leads to electrostatic repulsion). pH value at which these transformations take place depending on the structure of anthocyanins [44]. Apparently, at pH 6, the desorption solution pH was insufficient for the loss of a positive electric charge by cyanidin 3-glucoside and peonidin 3-glucoside. Therefore, they remained in the gel phase of CJMC-5, CEM Type-II, and CSE-fg membranes but were desorbed from the central part of the large macropores of the MK-40 membrane. We will discuss this phenomenon in Section 3.3.3 in more detail.

#### 3.3.2. Effect of Grinding

**Figure 12:**
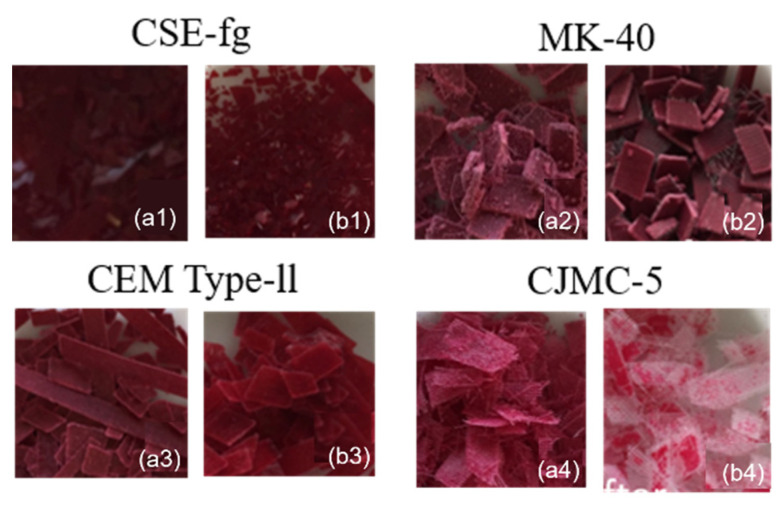
Photos of the grinded membranes (**a1**–**a4**) before and (**b1**–**b4**) after treatment in 25% acetonitrile/25% methanol/25% isopropanol/25% water at pH 6.

Cryomill grinding of membranes (Protocol 1) resulted in an increase of the ion-exchange material area in contact with the desorption solution (Figure 12). Consequently, this should have led to polyphenol extraction enhancement from the membrane material. However, if, after grinding CSE-fg coupons, the anthocyanin concentration in the solution increased after soaking, their concentrations decreased after grinding and soaking in the case of MK-40, CJMC-5, and CEM Type-II coupons. PACs were not extracted, as in the case of entire coupons for CJMC-5 and CEM Type-II, while grinding the MK-40 membrane before the desorption stage led to a decrease in their concentrations in the desorption solution. This result, paradoxical at first glance, was easy to explain, considering the data of the optical microscopy (Table 6, Figure 7).

The sorption/desorption processes of the CJMC-5 membrane were controversial. On the one hand, this thin membrane had macropores, which ensured unhindered penetration of anthocyanins into its volume and diffusion back from the membrane (Figure 6). Hence, grinding coupons and soaking in a mixture of solvents led to an almost complete removal of colored polyphenols from CJMC-5 (Figure 12b). On the other hand, anthocyanins cannot be detected after grinding the coupons. Apparently, the main reason was the too low concentration of these substances in the membrane, which was less than the limit of quantification of the method used to determine anthocyanin concentration. In contrast, after 168 h of soaking in cranberry juice, the entire volume of CSE-fg, which was the thinnest membrane, was filled with anthocyanins (Figure 8), even though this membrane did not contain large pores. As mentioned above, this was explained by its high ion-exchange capacity, promoting electrostatic interactions with polyphenols of cranberry juice at pH 2.45, and the high affinity of its aromatic matrix to the aromatic structure of anthocyanins (Figure 11), promoting π-π (staking) interactions. Therefore, an increase in the contact area of CSE-fg with the solvents mixture due to membrane grinding increased the concentration of anthocyanins in the desorption solution. Concerning the MK-40 membrane, part of this thick membrane did not contain any polyphenols even after 168 h of soaking in cranberry juice. The grinding of the coupons exposes these areas in the cross-sections of the MK-40 (Table 6). Therefore, polyphenols, removed by the solvent mixture from the surface and subsurface layers, were again partially sorbed by the areas free of polyphenols. This phenomenon was so significant that it led to a clarification of the solvent mixture after soaking the MK-40 grinding coupon in comparison with the entire coupon (Figure 10b and Figure 12b). A similar phenomenon, apparently, took place in the case of CEM Type-II, which had almost no large macropores and where the diffusion of anthocyanin molecules into which was restrained due to the small amount of macropores.

#### 3.3.3. Effect of Finer Grinding by Homogenizer

Ultrafine grinding step (Protocol 2), prior to desorption in the mix of solvents, should have reduced steric hindrances arising during the extraction of polyphenols from membranes. As presented in Table 7 for PAC content, ultrafine grinding by homogenization resulted in the appearance of polymers (30.2 ± 0.5 μg PACs/cm^3^ membrane) that could not be extracted from entire and cryogenic grinding MK-40 coupons. Moreover, while PACs (monomers, 2mers, and polymers) were detected in the case of the CSE-fg membrane, a decrease in the concentration of monomers, 2mers, 3mers, and 4mers was observed for MK-40 coupon. In addition, for both membranes’ anthocyanin concentrations decreased (Table 8) after the stage of ultrafine grinding in comparison with entire and cryogenic grinding. Apparently, PACs monomers, 2mers, 3mers, and 4mers, as well as anthocyanins, were partially sorbed by membrane meso- and micropores, into which they could not penetrate before the coupons were ground finer. Polymers, on the contrary, were formed in meso-, macropores, and structural defects of MK-40 and CSE-fg membranes due to the association of PACs monomers. The opening of such pores and defects results in the extraction of these substances with a mixture of solvents.

#### 3.3.4. Effect of pH of the Desorption Solution

The effect of the pH adjustment (pH 10) of the desorption solution was evaluated to determine if it enhanced the desorption and the recovery of PACs and anthocyanins, which were adsorbed by membranes due to electrostatic interactions.

The use of a mixture of solvents at pH 10 increased the concentration of all types of anthocyanins in the desorption solution in the case of MK-40 and CSE-fg membranes (Table 8). Moreover, for desorption at pH 10, the MK-40 presented higher concentrations of each anthocyanin, in comparison with CSE-fg. In addition, cyanidin 3-glucoside and peonidin 3-glucoside were extracted from the CSE-fg coupon, which remained in the membrane if pH of the desorption solution was 6.

As already discussed above, the flavilyum ion of anthocyanins was positively charged at the pH of the cranberry juice (2.45 ± 0.02) [44]. Then, it can interact with the negatively charged sites of the cation-exchange membrane by electrostatic interactions, which caused membrane fouling [5]. Hence, when the pH value of the mixture of solvents was 6, anthocyanin cations should have lost their electrical charge and turned into neutral molecules. However, the pH value inside the membrane was 1–3 units lower than pH of the external solution (Table 5) due to Donnan’s exclusion of protons from the cation-exchange membranes—products of protolysis of anthocyanins [21]. At such pH values, some of the anthocyanins remained cations and cannot be desorbed from the membranes. Increasing the pH value of the desorption solution to 10 solved this problem. Anthocyanins acquired a negative charge, and consequently, the electrostatic repulsion of these anions from the negatively charged fixed groups facilitated the extraction of these substances from the membranes [21].

Regarding PAC content (Table 7), an increase in the pH of the desorption solution promoted the extraction of not only anthocyanins but also PACs 2mers and 3mers. Moreover, the monomer concentrations do not statistically change in the case of pH 6 or 10. It can be attributed to the ability of PACs oligomers to partially degrade in an alkaline environment [45]. The reduction of the number of PACs of similar units would lead to a decrease in the size of PACs and would facilitate their extraction from the membranes.

## 4. Conclusions

This study aimed to better understand the mechanisms underlying the adsorption and desorption of PACs and anthocyanins from cranberry juice on pseudohomogeneous and heterogeneous cation-exchange membrane to prevent organic fouling. The main phenomena that can affect the sorption of polyphenols (PACs and anthocyanins) from cranberry juice by ion-exchange membranes, as well as an estimate of the probability of the contribution of these phenomena, considering the characteristics of the studied membranes, are presented in Table 9. Based on these main phenomena, results showed that some membranes (MK-40, CEM Type-II, and CSE-fg) were more prone to fouling mainly due to (1) their high ion-exchange capacities, (2) their lower thickness and (3) the presence of meso- and macropores in their matrices that reached the surface. Indeed, at the pH of the juice, high IECs led to electrostatic interactions between polyphenolic ions (e.g., the flavilyum ion of anthocyanins is positively charged at pH 2.45) and the fixed groups of the membrane (-SO_3_-). As demonstrated here, for the same anthocyanin, results were in relation to the ion-exchange capacity of each membrane. However, π-π (stacking) interactions between anthocyanins and the aromatic matrix in some way counteracted the extraction of these polyphenols (from MK-40 and CSE-fg) if polar aliphatic desorption solutions were used. In addition, when the pH of the desorption solution was increased up to 10, hydroxide ions competed with the membrane and polyphenols, consequently removing these compounds from the membranes by inducing the transformation of anthocyanins into neutral molecules or anions and weakening the electrostatic interactions of anthocyanins with the fixed groups of the membranes. For PACs, which are polymers of catechins, such an increase in pH would partially degrade them, leading to a decrease in their size and facilitating their extraction from the membranes. Concerning thickness, the fact that the MK-40 membrane (the thickest membrane) presented a lower reduction in its electrical conductivity, although it had one of the highest IEC, was due to the length of the path polyphenols must cover to fill the entire volume of the membrane. In addition to both previous membrane characteristics, the presence or absence of extended macropores that reached the surface of the membrane may affect the fouling by polyphenols. Indeed, these macropores facilitated the diffusion delivery of high molecular weight substances into the membrane volume by removing or decreasing the steric hindrances. The anthocyanins or small polyphenols can primarily diffuse in the macropores, localized in the threads of the reinforcing cloth, and then spread to the nearby space due to the pores between the resin particles and the inert binder. Hence, for the CJMC-5 membrane, the combination of large pores of ion-exchange material with extended pores around the threads of the reinforcing fabric membranes allowed a faster transfer of anthocyanin cations. Therefore, the entire volume of this membrane was fouled with anthocyanins, and although having a lower exchange capacity, the CJMC-5 was more affected by the anthocyanins cations, resulting in a drastic reduction of its electrical conductivity. Concerning PACs and macropores, due to their large sizes, they can be sorbed on the surface of the membranes or located in the macropores. Hence, during desorption from the entire membranes, PACS were removed only from the MK-40 membrane due to the presence of such macropores.

Finally, concerning desorption protocols, it appeared that the pH adjustment of the desorption solution to pH 10 allowed a better recovery of anthocyanins, confirming the sorption of polyphenols, mainly anthocyanins, by electrostatic interactions inside the membrane matrix. While the desorption of the entire membrane in the initial solution without pH adjustment (pH 6) allowed a better extraction of PACs, confirming the fact that the sorption of PACS is mainly at the surface of the membrane or in the macropores by π-π stacking between the aromatic ring of the polyphenols and the polymer. These results are of great interest for the use of a cation-exchange membrane in the fruit juice and wine industries.

## Figures and Tables

**Table 1:** Physico-chemical characteristics of cranberry juice.

**pH**	2.45 ± 0.02
**°Brix**	17.8 ± 0.1
**Conductivity (µS/cm)**	3817.3 ± 20.2
**Proanthocyanidins (ppm)**	
**Monomers**	87.8 ± 1.1
**2-mers**	202.3 ± 16.0
**3-mers**	87.2 ± 3.6
**4-mers**	40.1 ± 1.2
**5-mers**	14.9 ± 1.2
**6-mers**	10.0 ± 0.7
**7-mers**	1.2 ± 2.1
**Polymers**	44.3 ± 16.9
**Anthocyanins (ppm)**	
**Cyanidine 3-galactoside**	78.7 ± 0.3
**Cyanidine 3-glucoside**	3.6 ± 0.1
**Cyanidine 3-arabinoside**	70.7 ± 0.4
**Peonidine 3-galactoside**	96.1 ± 0.5
**Peonidine 3-glucoside**	7.5 ± 0.3
**Peonidine 3-arabinoside**	47.4 ± 0.4

**Table 2:** Characteristics of membranes under study.

Membrane	Ion Exchange Matrix	Macropores	Fixed Groups	Reinforcing Cloth
**CSE-fg**	Aromatic DVB+PS	Absence	-SO_3_H	PVC fabric
**MK-40**	Aromatic DVB+PS	Presence	-SO_3_H	nylon mesh
**CEM Type-II**	Aliphatic polyamide	Absence	-SO_3_H	3D polyolefin fibers structure
**CJMC-5**	Aliphatic PVDF+SSS	Presence	-SO_3_H	polyester mesh

DVB+PS is a copolymer of polystyrene and divinylbenzene; PE is low-pressure polyethylene; PA is polyamide; PVC is polyvinyl chloride, PVDF is polyvinylidene fluoride. The side chains of CJMC-5 matrix are self-crosslinking with cross-linking sodium 4-styrenesulfonate (SSS) agent [19].

**Table 3:** The thickness of membranes according to NaCl concentration.

Membrane	Thickness (μm)				
	0.10 M	0.25 M	0.50 M	0.75 M	1.00 M
**CSE-fg**	142 ± 3 ^dAB^	136 ± 4 ^cA^	142 ± 3 ^dAB^	137 ± 4 ^dA^	145 ± 4 ^dB^
**MK-40**	525 ± 11 ^aAB^	518 ± 11 ^aAB^	532 ± 15 ^aA^	514 ± 6 ^aB^	530 ± 7 ^aAB^
**CEM Type-II**	179 ± 4 ^bA^	170 ± 5 ^bB^	175 ± 3 ^bAB^	169 ± 3 ^bB^	176 ± 3 ^bC^
**CJMC-5**	154 ± 3 ^cA^	145 ± 5 ^cB^	154 ± 2 ^cA^	145 ± 3 ^cB^	153 ± 4 ^cA^

Lowercase: Data in the same column with different letters (a–d) are significantly different (Tukey, *p* < 0.05). Uppercase: Data in the same line with different letters (A–D) are significantly different (Tukey, *p* < 0.05).

**Table 4:** The volume fraction of the intergel space of membranes under study and corresponding electrical conductivity ($\bar{\kappa }$) and ion exchange capacity ($\bar{Q}$) of their gel phases.

Matrix	Membrane	f_2_	$\bar{\kappa }$	$\bar{Q}$
**Aromatic**	**CSE-fg**	0.073 ± 0.030	6.9 ± 0.8	1.73 ± 0.05
	**MK-40**	0.243 ± 0.010	3.8 ± 0.3	1.88 ± 0.03
**Aliphatic**	**CEM Type II**	0.139 ± 0.010	1.8 ± 0.1	1.57 ± 0.03
	**CJMC-5**	0.335 ± 0.060	1.2 ± 0.3	0.88 ± 0.08

**Table 5:** pH of the external and internal CJMC-5 membrane solution according to the color indication scale (Figure 6d).

External Solution	Internal Solution
3.6	2.7
6.9	4.0
9.3	7.0

**Table 6:** Evolution of the MK-40 membrane thickness and respective anthocyanin-free layer thickness in its cross-section, depending on the soaking duration in cranberry juice.

Membrane Soaking Time in Cranberry Juice (h)	Membrane Thickness (μm)	Thickness of the Anthocyanins-Free Layer (µm)
3	521 ± 2	370 ± 5
17	523 ± 2	350 ± 5
72	532 ± 2	270 ± 5
168	549 ± 2	170 ± 5

**Table 7:** Proanthocyanidin content of desorption solutions (DS) (µg PACs/cm^3^ membrane).

PAC	Desorption Method	CSE-fg	MK-40	CEM Type-II	CJMC-5
**Monomers**	Entire, DS pH 6	ND ^bA^	76.4 ± 2.9 ^aA^	ND ^bA^	ND ^bA^
	Cryogenic grinding, DS pH 6	ND ^bA^	55.0 ± 12.8 ^aB^	ND ^bA^	ND ^bA^
	Cryogenic grinding, DS pH 10	89.6 ± 5.6 ^bB^	62.1 ± 5.9 ^aAB^	-	-
	Cryogenic + finer grinding, DS pH 6	95.6 ± 3.6 ^bB^	57.2 ± 2.4 ^aB^	-	-
**2 mers**	Entire, DS pH 6	ND ^bA^	375.0 ± 14.7 ^aA^	ND ^bA^	ND ^bA^
	Cryogenic grinding, DS pH 6	ND ^bA^	210.0 ± 91.2 ^aB^	ND ^bA^	ND ^bA^
	Cryogenic grinding + pH 10	80.3 ± 5.3 ^bB^	226.4 ± 21.0 ^aB^	-	-
	Cryogenic + finer grinding, DS pH 6	39.4 ± 1.5 ^bC^	16.9 ± 0.7 ^aC^	-	-
**3 mers**	Entire, DS pH 6	ND ^bA^	39.8 ± 11.5 ^aA^	ND ^bA^	ND ^bA^
	Cryogenic grinding, DS pH 6	ND ^bA^	13.9 ± 8.0 ^aBC^	ND ^bA^	ND ^bA^
	Cryogenic grinding, DS pH 10	ND ^bA^	17.4 ± 1.9 ^aBC^	-	-
	Cryogenic + finer grinding, DS pH 6	ND ^bA^	6.3 ± 0.6 ^aC^	-	-
**4 mers**	Entire, DS pH 6	ND ^bA^	12.3 ± 2.2 ^aAB^	ND ^bA^	ND ^bA^
	Cryogenic grinding, DS pH 6	ND ^bA^	3.0 ± 5.2 ^aB^	ND ^bA^	ND ^bA^
	Cryogenic grinding, DS pH 10	ND ^aA^	ND ^aC^	ND ^aA^	ND ^aA^
	Cryogenic + finer grinding, DS pH 6	ND ^aA^	ND ^aC^	ND ^aA^	ND ^aA^
**Polymers**	Entire, DS pH 6	ND ^aA^	ND ^aA^	ND ^aA^	ND ^aA^
	Cryogenic grinding, DS pH 6	ND ^aA^	ND ^aA^	ND ^aA^	ND ^aA^
	Cryogenic grinding, DS pH 10	ND ^aA^	ND ^aA^	-	-
	Cryogenic + finer grinding, DS pH 6	98.9 ± 87.8 ^aB^	30.2 ± 0.5 ^aB^	-	-

-: Not analyzed. ND: not detected. Uppercase (A–C) indicate significant differences amongst desorption method within the same membrane and proanthocyanidin (Tukey, *p* < 0.05). Lowercase (a–b) indicates significant differences amongst the four membranes within the same proanthocyanidin and the same desorption method (Tukey, *p* < 0.05).

**Table 8:** Anthocyanin content of desorption solutions (DS) (µg anthocyanin/cm3 membrane).

Anthocyanin	Desorption Method	CSE-fg	MK-40	CEM Type-ll	CJMC-5
**Cyanidin 3-** **galactoside**	Entire, DS pH 6	24.5 ± 2.9 ^dA^	74.4 ± 7.5 ^aA^	88.4 ± 47.7 ^aA^	4.6 ± 8.0 ^bA^
	Cryogenic grinding, DS pH 6	68.9 ± 2.6 ^aB^	47.7 ± 10.5 ^aB^	48.7± 13.9 ^aA^	ND ^bB^
	Cryogenic grinding, DS pH 10	341.0 ± 29.3 ^bC^	470.6 ± 30.1 ^aC^	-	-
	Cryogenic + finer grinding, DS pH 6	21.3 ± 2.5 ^bA^	10.9 ± 2.4 ^aD^	-	-
**Cyanidin 3-** **glucoside**	Entire, DS pH 6	ND ^bA^	4.1 ± 0.4 ^aA^	ND ^bA^	ND ^bA^
	Cryogenic grinding, DS pH 6	ND ^bA^	0.10 ± 0.01 ^aB^	ND ^bA^	ND ^bA^
	Cryogenic grinding, DS pH 10	13.8 ± 1.6 ^bB^	18.1 ± 1.0 ^aC^	-	-
	Cryogenic + finer grinding, DS pH 6	ND ^bA^	0.8 ± 0.2 ^aB^	-	-
**Cyanidin 3-** **arabinoside**	Entire, DS pH 6	29.0 ± 5.7 ^dA^	76.0 ± 8.2 ^aA^	124.3 ± 63.8 ^aA^	5.0 ± 8.6 ^bA^
	Cryogenic grinding, DS pH 6	73.8 ± 3.7 ^cB^	39.4 ± 7.7 ^aB^	52.7 ± 11.5 ^aA^	ND ^bB^
	Cryogenic grinding, DS pH 10	405.9 ± 27.7 ^bC^	504.7 ± 25.5 ^aC^	-	-
	Cryogenic + finer grinding, DS pH 6	12.0 ± 11.1 ^aA^	8.9 ± 1.7 ^aD^	-	-
**Peonidin 3-** **galactoside**	Entire, DS pH 6	32.2 ± 1.8 ^bA^	83.2 ± 10.3 ^aA^	108.1 ± 57.9 ^aA^	7.4 ± 12.8 ^b*^
	Cryogenic grinding, DS pH 6	89.5 ± 5.7 ^bB^	62.8 ± 16.9 ^abA^	53.7 ± 10.2 ^aA^	ND ^c^
	Cryogenic grinding, DS pH 10	338.8 ± 35.5 ^bC^	520.9 ± 29.8 ^aB^	-	-
	Cryogenic + finer grinding, DS pH 6	27.7 ± 3.8 ^bA^	1.5 ± 0.3 ^aC^	-	-
**Peonidin 3-** **glucoside**	Entire, DS pH 6	ND ^bA^	10.2 ± 0.8 ^aA^	ND ^bA^	ND ^bA^
	Cryogenic grinding, DS pH 6	ND ^bA^	7.0 ± 1.8 ^aB^	ND ^bA^	ND ^bA^
	Cryogenic grinding, DS pH 10	28.5 ± 2.9 ^bB^	43.9 ± 2.7 ^aC^	-	-
	Cryogenic + finer grinding, DS pH 6	ND ^bA^	15.7 ± 3.1 ^aD^	-	-
**Peonidin 3-** **arabinoside**	Entire, DS pH 6	26.0 ± 3.4 ^acA^	46.4 ± 6.3 ^aA^	82.3 ± 40.3 ^aA^	3.3 ± 5.7 ^bA^
	Cryogenic grinding, DS pH 6	52.5 ± 3.6 ^cB^	28.3 ± 6.8 ^aB^	33.0 ± 9.0 ^aA^	ND ^bB^
	Cryogenic grinding, DS pH 10	213.8 ± 20.0 ^bC^	294.2 ± 15.5 ^aC^	-	-
	Cryogenic + finer grinding, DS pH 6	16.7 ± 3.4 ^bD^	7.3 ± 1.6 ^aD^	-	-

ND: Not detected. -: Not analyzed. Uppercase (A–D) indicates significant differences amongst desorption method within the same membrane and proanthocyanidin (Tukey, *p* < 0.05). Lowercase (a–c) indicates significant differences amongst the four membranes within the same proanthocyanidin and the same desorption method (Tukey, *p* < 0.05).

**Table 9:** Main phenomena affecting the sorption of polyphenols from cranberry juice on membranes.

Phenomenon	Probability of Impact on Sorption Capacity			
	CSE-fg	MK-40	CEM Type-II	CJMC-5
**Electrostatic interactions** between polyphenolic ions and fixed groups of membranes (depends on the exchange capacity of the membranes)	high	moderate	moderate	low
**Hydrophobic-hydrophobic π-π (stacking) interactions** of aromatic rings of polyphenols and membrane’s polymers	high	high	negligible	low
**Ion-dipole** (hydrogen bonds) **and dipole-dipole** (Van der Waals) **interactions**. Hydrogen bonds between –OH and/or carbonic groups of polyphenols with oxygen of the sulfonic fixed groups as well as hydrogen of the aliphatic chains of materials that are part of the membrane (PA, PVC, nylon, etc.)	moderate	moderate	high	high
**Steric limits on the transport of large particles** (depends on the presence of macropores and their localization)	high	low	moderate	negligible

## Data Availability

Not applicable.
